# Role of Human Endogenous Retroviral Long Terminal Repeats (LTRs) in Maintaining the Integrity of the Human Germ Line

**DOI:** 10.3390/v3060901

**Published:** 2011-06-21

**Authors:** Meihong Liu, Maribeth V. Eiden

**Affiliations:** Laboratory of Cellular and Molecular Regulation, National Institute of Mental Health, 49 Convent Drive MSC 4483, Bethesda, MD 20892, USA; E-Mail: lmeihong@mail.nih.gov

**Keywords:** genomic stability, retroelement, primate evolution

## Abstract

Retroviruses integrate a reverse transcribed double stranded DNA copy of their viral genome into the chromosomal DNA of cells they infect. Occasionally, exogenous retroviruses infect germ cells and when this happens a profound shift in the virus host dynamic occurs. Retroviruses maintained as hereditable viral genetic material are referred to as endogenous retroviruses (ERVs). After millions of years of co-evolution with their hosts many human ERVs retain some degree of function and a few have even become symbionts. Thousands of copies of endogenous retrovirus long terminal repeats (LTRs) exist in the human genome. There are approximately 3000 to 4000 copies of the ERV-9 LTRs in the human genome and like other solo LTRs, ERV-9 LTRs can exhibit distinct promoter/enhancer activity in different cell lineages. It has been recently reported that a novel transcript of p63, a primordial member of the p53 family, is under the transcriptional control of an ERV-9 LTR [1]. The expression of different *p63* transcript isoforms has been previously shown to have an important role in replenishing cutaneous epithelial stem cells and maintaining the fidelity of the female germ line [2]. In this recent report, a novel p63 transcript, designated GTAp63, is described as specifically expressed in healthy human testes and germ cell precursors of human testes but not in testicular cancer cells. The ability of ERV-9 regulatory regions to contribute to the maintenance of male germ line stability is yet another example of how ERVs have evolved to serve an important function in the physiology of their human hosts.

The complete sequencing of the human genome produced an unexpected windfall—the discovery that genetic elements associated with endogenous retroviruses (ERVs) far outnumbered protein-encoding genes. The transcriptional and transpositional activities of ERVs would predict destabilization of a metazoan genome with such a high retroviral burden. Therefore, it is not surprising that in the millions of years since ERVs first established residence in the human genome most have been rendered inactive, through epigenetic suppression, recombination or mutation. Replication-competent retroviruses integrated into the host genome are flanked by long terminal repeats (LTRs). LTRs contain regulatory elements essential for gene expression including promoters, enhancers and polyadenylation sequences as well as sequences necessary for retrotransposition. Among human ERVs (HERVs) that retain some degree of function, several have acquired physiologically relevant roles in their hosts. For example, one of several integrants of the human retrovirus HERV-E contributes enhancer activity to the human amylase gene that allows for salivary gland-specific regulation [3]. HERV LTRs have also been proposed to regulate expression of the apolipoprotein-C1 gene in liver, and the endothelin-B receptor and pleiotrophin genes in placenta [4]. The role of endogenous LTRs as promoters for other human genes has been recently reviewed [5]. Here, we comment on some intriguing new developments reported by Beyer *et al.* [1] on the role of ERV-9 LTRs in gene expression in human male germ cells.

## 1. ERV-9 LTR Driven Human Transcripts

ERV-9 LTRs are found only in the primate genome where they have been maintained for at least 15 million years [6]. One feature that distinguishes ERV-9 LTRs from other endogenous human LTRs is the presences of 14 tandem repeat elements that contain recurrent CCAAT, GTGGGGA and GATA nucleotide motifs. These DNA motifs bind transcription factors that are expressed preferentially in hematopoietic progenitor cells [7] and in reproductive tissues [8–11]. ERV-9 LTR complexes formed by the competitive recruitment of these transcription factors have been reported to modulate human globin gene switching, selectively transferring these transcription factors 70 kilobases away to activate transcription of the β-globin gene in hemapoietic progenitor cells [12].

Since ERV-9 transcription factors are highly evolutionarily conserved, ERV-9 LTR (referred to herein as globin ERV-9 LTR) driven GFP gene expression was evaluated in transgenic zebrafish and the distribution of ERV-9 initiated transcripts in zebrafish was compared to those found in humans [13]. Using a globin ERV-9 specific sequence probe for *in situ* hybridization analysis, ERV-9 initiated gene expression in transgenic zebrafish and in humans occurs in both oocytes and various progenitor cells but not testes [13]. The identity of the genes transcribed under the influence of this ERV-9 promoter activity in oocytes has not been determined.

## 2. TAp63 Isoforms Present in the Human p63 Locus

**Figure 1.**
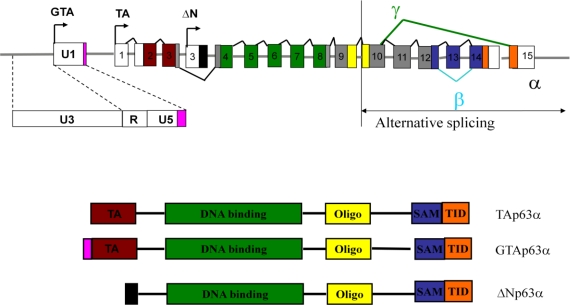
Genomic organization of human *p63* locus. The *p63* locus contains more than one promoter. Arrows designate the transcription initiation sites for the TAp63 (TA) and ΔNp63 (ΔN) as well as GTAp63 (GTA) transcripts from its distinct ERV-9 promoter. Alternate splicing within the 3′ region of the gene results in the formation of the α, β and γ isoforms of the TAp63 and ΔNp63. The main structural domains include the N-terminal transactivation domain (TA), specific to the TAp63α and GTApTAp63α isoforms, DNA binding (green), oligomerization (yellow), SAM, or sterile alpha motif, a protein-protein interaction domain (purple) and the TID transactivation inhibitory domain (orange) [16]. The promoter located within the U3 of an ERV-9 LTR initiates transcription of GTAp63. The coding region corresponding to the 19 residues in the GTAp63 protein derived from the ERV-9 U5 is shown in pink.

From 1979 to the present the tumor suppressor *p53* has been extensively studied [14]. Twenty years after the initial reports on *p53*, another member of this gene family, *p63*, emerged as a gene with distinct developmental properties. The p63 gene encodes two transcript isoforms, ΔNp63 and TAp63. ΔNp63 and TAp63 have unique promoters and each encode three variants designated α, β, and γ resulting from alternate 3′ splicing events (Figure 1). The major difference between the ΔNp63 and TAp63 isoforms is the presence of a unique N-terminal transactivating (TA) domain in the TAp63 isoform. ΔNp63α is the major functioning isoform of ΔNp63 and is abundantly expressed in the immature squamous epithelium and basal or reserve cells derived from breast, salivary gland, prostate, and cervix [2]. The expression of ΔNp63α is required for the survival and maintenance of progenitor cells of stratified squamous epithelia [2]. TAp63 functions as a tumor suppressor inducing apoptosis activities similar to those of the p53 protein. Just as p53 monitors and suppresses the initiation of tumorigenesis in the genome of somatic cells, TAp63 appears to survey the female germ line for DNA damage. Using TAp63 isoform-specific antibodies it was concluded that the TAp63 isoform predominantly expressed in oocytes is TAp63α. TAp63α was not detected in the testes of newborn or adult mice suggesting TAp63α mediated germ line protection is specific to female mice [15].

## 3. GTAp63 is Expressed in Male Germ Cells

The report recently published by Beyer *et al.* describes a unique role of an ERV-9 LTR in regulating expression from the p63 gene [1]. These investigators discovered that the ERV-9 LTR located upstream of the *TAp63* gene on chromosome 3 promotes the tissue-specific expression of a unique transcript encoding **g**erm cell-associated **t**ranscriptionally **a**ctive p63 (**GTA**p63). GTAp63, in contrast to TAp63, is expressed at high levels in spermatogenic precursors, but its expression is extinguished in mature sperm. GTAp63 is expressed in the diploid spermatogonia of healthy human testis, but not in testicular cancer cells. Caspase cleavage of the GTAp63 protein in response to DNA damage removes the transcription-inhibitory SAM and TID domains of p63, thereby generating p63 proteins with greater transcriptional activity resulting in an enhanced proapoptotic response. This suggests a role for GTAp63 in protecting the genomic integrity of the human male germ line. What is unusual about this employment of ERV-9 as an ancillary *p63* gene promoter is that a portion of ERV-9 is incorporated in the GTAp63 protein. ERV-9 like all retroviral LTRs contains U3, R and U5 regions. The R region and U5 regions are typically 5′ untranslated regions in viral transcripts as well as in human genes initiated by ERV LTRs. ERV-9 initiated GTAp63 transcripts, in contrast, yield a protein that contains a 19 residue N-terminal segment derived from the U5 region of the ERV-9 LTR itself. GTAp63 is therefore a novel previously unobserved class of mammalian protein representing an expressed virus-host chimera.

The discovery by Beyer *et al.* that an ERV-9 LTR initiates transcription of GTAp63 proteins that protect the integrity of the male germ line [1], represents a convergence of the two independent lines of scientific investigation; the transcription of human genes by ERV-9 LTRs and the function of *p63* gene products in germ cells. Distinct ERV-9 LTRs control the spatial and temporal regulation of transcripts in male germ cells [1] and oocytes [13] and GTAp63α and TAp63α function to maintain the fidelity of the male and female germ line, respectively [1,2,15]. Clearly it will be important to resolve the basis for the observed differences in p63- and globin-ERV-9 initiated transcription, in human testes and oocytes. Antibodies and probes specific for the GTAp63a that distinguish its expression from TAp63a will be of great value in delineating the distinctive role of these two proteins in the human line.
